# Effects of Kudiezi Injection on Serum Inflammatory Biomarkers in Patients with Acute Cerebral Infarction

**DOI:** 10.1155/2018/7936736

**Published:** 2018-09-02

**Authors:** Xuemei Liu, Xianglan Jin, Baoxin Chen, Xiaohan Liu, Xiao Liang, Xiaolei Fang, Hongyun Wu, Xiaoyu Fu, Hong Zheng, Xiao Ding, Na Duan, Yunling Zhang

**Affiliations:** ^1^Central Laboratory, Dongfang Hospital, Beijing University of Chinese Medicine, Beijing, China; ^2^Department of Neurology, Dong Fang Hospital, Beijing University of Chinese Medicine, Beijing, China; ^3^Beijing University of Chinese Medicine, Beijing, China; ^4^Department of Emergency, Dong Fang Hospital, Beijing University of Chinese Medicine, Beijing, China; ^5^Department of Neurology, Affiliated Hospital of Shandong University of Traditional Chinese Medicine, Jinan, China; ^6^Department of Neurology, Beijing Huairou District Hospital of Chinese Medicine, Beijing, China; ^7^Xiyuan Hospital, China Academy of Chinese Medical Sciences, Beijing, China

## Abstract

**Background:**

Kudiezi injection is a traditional Chinese medicine for acute cerebral infarction, but the exact mechanisms are poorly understood.

**Objective:**

To investigate the mechanisms of Kudiezi injection on the inflammatory response in the treatment of acute cerebral infarction.

**Methods:**

This was a prospective study of patients with acute cerebral infarction within 48 h of onset and treated between July 2012 and July 2016 at three hospitals in China. The patients were randomized to routine treatments (control group) versus routine treatments and Kudiezi injection (Kudiezi group). The National Institutes of Health Stroke Score was assessed on days 1, 3, 5, 7, and 14. The patients were tested for serum levels of pro- and anti-inflammatory cytokines (S100 calcium-binding protein B, neuron-specific enolase, interleukin-6, interleukin-10, interleukin-18, and matrix metaloproteinase-9; by enzyme-linked immunosorbent assay) immediately after admission and on days 3, 5, and 14.

**Results:**

Stroke scores were improved in both groups from days 1 to 14. On days 5 and 7, stroke scores in the Kudiezi group were lower than in the control group (*P* < 0.05). Compared with controls, the Kudiezi group had lower serum S100 calcium-binding protein B on day 14; higher interleukin-6 and interleukin-10 on day 3; lower interleukin-6 and interleukin-18 on day 5; and lower interleukin-18 and matrix metaloproteinase-9 on day 14.

**Conclusion:**

Kudiezi injection could lead to early reduction of interleukin-6, interleukin-18, matrix metaloproteinase-9, neuron-specific enolase, and S100 calcium-binding protein B levels and increases of interleukin-10 levels in patients with acute ischemic stroke. This trial is registered with ClinicalTrials.gov NCT01636154.

## 1. Introduction

Inflammation plays a key role in the pathogenesis of acute cerebral infarction and leads to brain damage [1, 2]. Numerous cytokines, both pro- and anti-inflammatory, are involved in this process. Interleukin- (IL-) 6 is one of the most important proinflammatory cytokines; it is expressed within a few hours after cerebral ischemia and is an acknowledged biomarker for long- and short-term neurological outcomes after ischemic stroke [3, 4]. IL-18 is a proinflammatory cytokine that stimulates the production of a large number of inflammatory factors after cerebral ischemia; it is also involved in brain edema and damage [5]. On the other hand, IL-10 is an anti-inflammatory cytokine that inhibits the inflammatory response, playing protective effects after stroke [6, 7].

Markers are also available to measure the extent of neurological damage after stroke. Indeed, changes of serum S100 calcium-binding protein B (S100B) levels can reflect the severity of neuroglial cell injury [8]. Serum neuron-specific enolase (NSE) is a specific indicator of brain neuronal damage and necrosis. High levels of serum S100B and NSE are associated with disease severity and poor patient prognosis [9, 10].

Kudiezi injection is the first-line traditional Chinese medicine (TCM) treatment for acute cerebral infarction and has been shown to improve the outcomes of patients with stroke [11–13]. The Kudiezi injection is a preparation made of *Ixeris sonchifolia* (Bge.) Hance and is made using the whole herb. In clinical practice, it is mainly used for the treatment of coronary heart disease, angina, and cerebral infarction. Its main components are amino acids, flavonoids, saponins, and sesquiterpene lactones [14–16]. Sesquiterpene lactones are considered as the major active compounds in Kudiezi injection because of their special structures and activities [15]. Nevertheless, the exact mechanisms of action of Kudiezi injection remain elusive. The protection mechanism of Kudiezi injection against ischemic reperfusion (I/R) cerebral damage may possibly be related to the biosynthesis of phenylalanine, tyrosine, and tryptophan [17]. KDZ protects the blood-brain barrier from disruption and improves cerebral outcomes following I/R by preventing the degradation of tight junction proteins, increasing caveolin-1 expression, and inhibiting p-caveolin-1 and p-Src, probably because of the ability of its main ingredients to bind to Src and inhibit its phosphorylation [18]. KDZ protects the brain against acute focal ischemic injury *in vivo* and *in vitro*. The underlying mechanisms might be associated with the anti-inflammatory effect of KDZ through the TLR4/NF-*κ*B signaling pathway [19]. Studies have shown that Kudiezi injection can reduce inflammatory markers such as IL-1*β*, IL-6, tumor necrosis factor (TNF)-*α*, C-reactive protein (CRP), toll-like receptor 4 (TLR4), and NADPH oxidase 4 (NOX4) in patients with acute cerebral infarction [12, 13, 20, 21]. Animal studies also showed that Kudiezi injection can downregulate the expression of nuclear factor (NF)-*κ*B in the cerebral cortex of rats with acute cerebral infarction and modulate the expression of a number of proteins involved in neuronal damage [20, 22]. Moreover, Kudiezi injection downregulates the expression of adhesion molecules (intercellular adhesion molecule- (ICAM-) 1 and vascular cell adhesion molecule- (VCAM-) 1) involved in the recruitment of inflammatory cells in human brain microvascular endothelial cells injured by high glucose concentrations [23]. In addition, the balance between oxidative stress and antioxidant capacity is involved in the extent of damage after ischemic stroke [24]. Kudiezi injection has been shown to shift the balance toward decreased oxidative stress in rats [25].

In order to improve our understanding of the mechanisms of Kudiezi injection in the treatment of stroke, patients with cerebral infarction within 48 h of onset were recruited to investigate the effects of Kudiezi injection on the inflammatory response during the treatment of acute cerebral infarction.

## 2. Study Subjects and Methods

### 2.1. Study Design and Patients

This was a prospective study of patients with acute cerebral infarction within 48 h of onset and treated between July 2012 and July 2016 (ClinicalTrials.gov NCT01636154). This study was approved by the Ethics Committee of Dongfang Hospital, Beijing University of Chinese Medicine.

The study patient population was from the Dongfang Hospital affiliated to Beijing University of Chinese Medicine, Affiliated Hospital of Shandong University of Traditional Chinese Medicine, and Beijing Huairou District Hospital of Chinese Medicine. The inclusion criteria were (1) diagnosis of acute cerebral infarction according to the “Chinese treatment guidelines of acute ischemic stroke 2010” [26]; (2) ≤48 h from onset; (3) National Institutes of Health Stroke Scale (NIHSS) scores of 5–25 points [27]; (4) male or female patients of ≥18 years of age; and (5) provided informed consent. The exclusion criteria were (1) cardiogenic brain embolism, cerebral infarction due to other causes, or unknown causes; (2) eligible and ready for thrombolytic therapy; (3) eligible and ready for endovascular treatment or had already undergone endovascular treatment; (4) severe systemic diseases of the heart, lung, liver, or kidney (alanine transaminase (ALT) or aspartate transaminase (AST) >2 times the upper limit of normal, creatinine > 1.5 times the upper limit of normal, acute attack of asthma or chronic obstructive pulmonary disease (COPD), or heart function of grade IV); (5) hemorrhage or tendency to bleed; (6) pregnant or lactating women; (7) limb dysfunction, mental disorders, or cognitive impairment affecting adequate patient evaluation; (8) allergy or contraindications to Kudiezi injection or its components; or (9) participated in another clinical trial within 3 months.

### 2.2. Grouping

Randomization was performed using a central computer system. The recruited patients were randomized to the routine treatment group (control group) and Kudiezi injection group (KDZ group).

### 2.3. Treatments

Treatments for improving cerebral blood circulation (including antiplatelet, anticoagulant, fibrinolysis, and dilation) and neuroprotective therapy were performed for patients in the control group according to the “Chinese treatment guidelines of acute ischemic stroke 2010” [26]. TCM injection, oral administration of TCM decoctions, and acupuncture were not performed.

The patients in the KDZ group received the same routine treatments as in the control group, as well as intravenous infusion of Kudiezi injection (Tonghua Huaxia Pharmaceutical Co. Ltd., Jilin, China). Kudiezi injection (40 ml in 250 ml of 0.9% sodium chloride) was intravenously injected once daily at 40 drops/min for 14 days.

### 2.4. Concomitant Medications

Drugs that had to be administrated due to comorbidities could remain unchanged, based on the physicians' experience. Any TCM that would affect the adequate evaluation of Kudiezi injection was prohibited.

### 2.5. Data Collection

Demographic data (age, gender, height, weight, previous disease history, drugs, smoking, and drinking) were collected. General physical examination (body temperature, breathing, resting heart rate, and blood pressure) was performed at admission.

### 2.6. Clinical Outcomes

NIHSS score was assessed on days 1, 3, 5, 7, and 14 [27]. Peripheral venous blood was sampled in the fasting state on days 1, 3, 5, and 14 to measure S100B, NSE, IL-6, IL-10, IL-18, and matrix metalloproteinase- (MMP-) 9 levels using commercial ELISA kits.

### 2.7. Blood Sampling

Peripheral venous blood (4 ml) was sampled from each patient in the fasting state, in the morning, on days 1, 3, 5, and 14 after admission. The blood was clotted at room temperature for 2 h and centrifuged at room temperature at 3000 rpm for 10 min. The supernatant was aliquoted (200 *μ*l/vial) and stored at −80°C. Repeated freeze thawing was avoided.

### 2.8. ELISA

S100B, NSE, IL-6, IL-10, IL-18, and MMP-9 were measured according to the kit's instructions. The absorbance of each well was measured at 450 nm using a microplate reader. The human IL-18 ELISA kit (D7620; R&D Systems, Minneapolis, MN, USA), human IL-10 ELISA kit (D1000B; R&D Systems, Minneapolis, MN, USA), human MMP9 ELISA kit (D6000; R&D Systems, Minneapolis, MN, USA), human S100B ELISA kit (DY1820-05; R&D Systems, Minneapolis, MN, USA), and human NSE ELISA kit (DENL20; R&D Systems, Minneapolis, MN, USA) were used.

### 2.9. Statistical Analysis

Statistical analysis was performed using the FAS population and GraphPad Prism version 6.0 (GraphPad Software Inc., San Diego, CA, USA). Categorical data were presented as frequency, percentage, or ratio and analyzed using the chi-square test. Continuous data were presented using means ± standard deviations (SD) and analyzed using the Student *t*-test. Two-sided *P* values < 0.05 were considered statistically significant.

## 3. Results

### 3.1. Characteristics of the Patients

**Figure 1:**
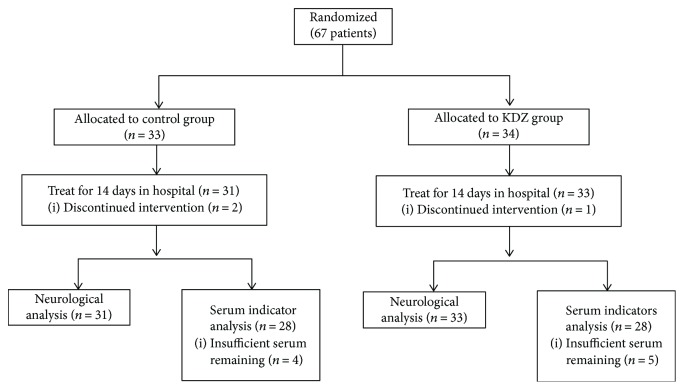
Patient flowchart.

Figure 1 presents the patient flowchart. Sixty-seven patients were randomized to the control (*n* = 33) and KDZ (*n* = 34) groups. Two patients discontinued intervention in the control group and one in the KDZ group because they refused to repeat blood sample collection. Neurological assessment was performed in 31 and 33 patients in the control and KDZ groups, respectively. After excluding those with missing data or blood samples, 28 patients were analyzed in each group. The baseline characteristics of the patients are shown in Table 1. There were no differences between the two groups regarding age, gender, risk factors, complications, and distribution of stroke subtypes (all *P* > 0.05).

### 3.2. Effects of Kudiezi Injection on Neurological Deficits

**Figure 2:**
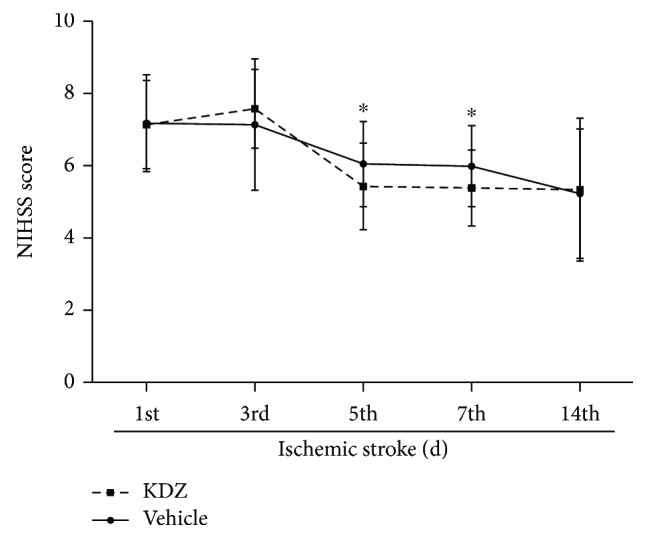
NIHSS score after acute ischemic stroke. NIHSS scores were decreased in both groups from day 1 to day 14. There were no differences between the two groups, except on days 5 and 7. *n* = 28/group. ^∗^*P* < 0.05 versus day 1. NIHSS: National Institutes of Health Stroke Scale.

In both groups, NIHSS score was improved from day 1 to day 14 (both *P* < 0.05). There were no differences in NIHSS scores between the two groups on days 1, 3, and 14 (all *P* > 0.05), but on days 5 and 7, NIHSS scores in the KDZ group were lower than in the control group (day 5: *P* = 0.030; day 7: *P* = 0.042) (Figure 2).

### 3.3. Effects of Kudiezi Injection on Serum S100B and NSE

**Figure 3:**
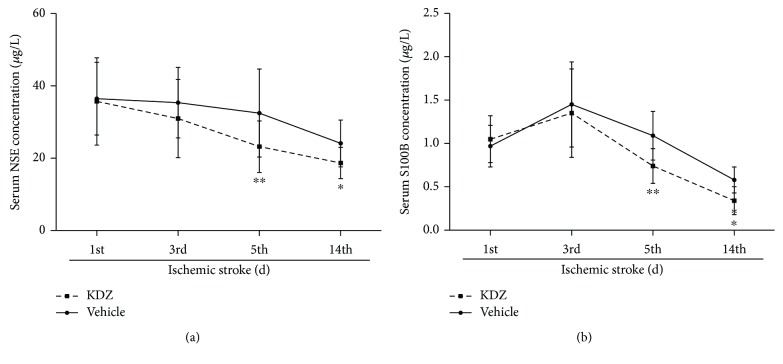
Serum NSE and S100B levels in acute ischemic stroke patients. (a) NSE levels. (b) S100B levels. *n* = 28/group. ^∗^*P* < 0.05, ^∗∗^*P* < 0.01, *t*-test, versus the control group at the same time point. NSE: neuron-specific enolase; S100B: S100 calcium-binding protein B; KDZ: Kudiezi injection.

NSE and S100B levels decreased in both groups from day 1 to day 14 (all *P* < 0.05). There were no differences of serum levels of NSE and S100B between the two groups on days 1 and 3 (all *P* > 0.05). From day 5, the KDZ group showed lower serum levels of NSE and S100B compared with controls (NSE: *P* = 0.001 and *P* = 0.0006; S100B: *P* < 0.0001 and *P* < 0.0001) (Figure 3).

### 3.4. Effects of Kudiezi Injection on Serum Inflammatory Factors

**Figure 4:**
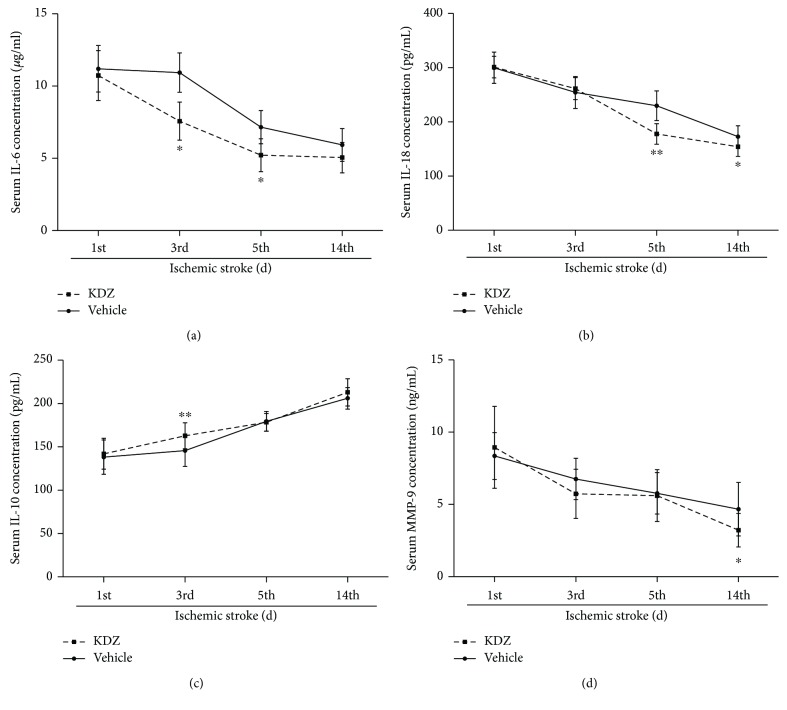
Serum inflammatory markers in acute ischemic stroke patients. (a) IL-6 levels. (b) IL-18 levels. (c) IL-10 levels. (d) MMP-9 levels. *n* = 28/group. ^∗^*P* < 0.05, ^∗∗^*P* < 0.01, *t*-test, versus the control group on the same time point. IL: interleukin; MMP: matrix metalloproteinase; KDZ: Kudiezi injection.

From day 1 to day 14, the levels of IL-6, Il-18, and MMP-9 decreased in both groups, while IL-10 levels increased (all *P* < 0.05). Compared with the control group, IL-6 levels were lower in the KDZ group on days 3, 5, and 14 (*P* < 0.0001, *P* < 0.0001, and *P* = 0.0006); IL-18 levels were lower in the KDZ group on days 5 and 14 (*P* < 0.0001 and *P* = 0.0005); IL-10 levels were higher in the KDZ group on day 3 (*P* = 0.0003); and finally, MMP-9 levels were lower on days 3 and 14 (*P* = 0.02 and *P* = 0.0009) (Figure 4).

### 3.5. Correlations

On day 5, the NIHSS scores were positively correlated with IL-18 (*r* = 0.329, *P* = 0.013) and S100B (*r* = 0.379, *P* = 0.004). On day 14, the NIHSS scores were negatively correlated with IL-10 (*r* = −0.447, *P* = 0.013). No other correlations were observed between NIHSS scores at different time points and biomarkers.

## 4. Discussion

Kudiezi injection is the first-line traditional Chinese medicine treatment for acute cerebral infarction, but its exact mechanisms of action are still poorly understood. Therefore, this study aimed to investigate the effects of Kudiezi injection on the inflammatory response in the treatment of acute cerebral infarction. The results showed that Kudiezi injection could significantly decrease serum IL-6, IL-18, MMP-9, NSE, and S100B levels and increase IL-10 levels in patients with acute stroke. These changes occurred earlier than with conventional stroke treatment.

S100B is mainly released by astrocytes [28]. Under physiological conditions, S100B is a neurotrophic cytokine that regulates intracellular signals, cell structure, and energy metabolism [29]. In addition, S100B is closely associated with glial cell proliferation, axonal growth, and calcium homeostasis, but excessive S100B levels lead to increased release of proinflammatory cytokines such as IL-6, IL-1*β*, and TNF-*α*. High S100B levels also lead to increased inflammatory stress-related enzymes such as inducible nitric oxide synthase and upregulated NF-*κ*B pathway, leading to neuronal death [30]. Hence, as a clinical biomarker, S100B, is highly sensitive for brain tissue damage. Indeed, in patients with mild brain injury, CT scan may be normal but serum S100B levels may be elevated significantly [31]. Elevated S100B is a marker of poor prognosis. In stroke, S100B is released in the cerebrospinal fluid at the beginning of the ischemic period and enters the bloodstream, where the levels peak within 24–120 h after ischemia [8]. NSE is a glycolytic enzyme found primarily in neuroendocrine cells and neuronal cytoplasm and is a marker of acute brain injury severity and clinical status [32]. NSE levels in the cerebrospinal fluid are elevated after cerebral ischemia. Serum NSE levels can be detected in the initial stage of the disease, peaking within 24–96 h after ischemia, and showing a decreasing trend when the symptoms disappear [33]. Serum NSE can be used as a marker of neurological function after stroke [34]. A previous animal study showed that Kudiezi injection could downregulate NF-*κ*B-related neuronal death after ischemic injury [19]. This could explain why NSE and S100B levels started to decrease earlier in the Kudiezi group than in the control group.

Serum levels of NSE and S100B in patients with acute cerebral infarction are positively correlated with the NIHSS [29]. In the present study, serum NSE and S100B in the KDZ group were decreased 5 days after admission and they remained lower than in the control group until the end of treatment (day 14), suggesting that Kudiezi injection can reduce the NSE and S100B levels. Although the NIHSS scores were not different between the two groups, Kudiezi injection could improve the patients' NIHSS score after 14 days of treatment, suggesting that it can be used to treat acute cerebral infarction and alleviate the symptoms of nerve injury of the patients. The lack of change of NIHSS could be because it is a somewhat suggestive and lacks sensitivity, as it is based on observations and not on quantitative measurements.

Inflammatory regulators (including proinflammatory and anti-inflammatory factors) play important roles in the pathogenesis of stroke. Inflammatory processes and injury-induced changes in neurotransmitter may further increase tissue damage [1, 35, 36]. IL-6 is a multifunctional factor produced by various types of cells that regulate immune responses, acute phase responses, and inflammation in general [37]. After cerebral ischemia, IL-6 levels increase and peak around 3 days after ischemia. IL-6 levels are closely associated with stroke severity, infarct volume, and poor prognosis [38, 39]. The results of the present study suggest that serum IL-6 levels in the KDZ group within 3–5 days after admission (i.e., 5–7 days after onset) were significantly lower compared to the control group.

In the brain, microglia and astrocytes are the main sources of IL-18 [40]. Elevated IL-18 levels after stroke are closely associated with ischemic brain injury [41] and increase with the aggravation of neurological impairments [42]. Elevated IL-18 levels in patients with acute cerebral infarction may be due to cytokine leakage from the infarct area but may also be from cerebrospinal fluid circulation [43]. In the present study, serum IL-18 levels in patients with acute cerebral infarction started to decrease after 5 days of Kudiezi injection and were still decreasing by day 14.

MMPs attack various extracellular matrixes. In the brain, they can promote neuronal cell death [44]. In the ischemic phase, MMP-9 is closely associated with the permeability and function of the blood-brain barrier [44]. In the early stages of cerebral ischemia (ranging from a few hours to a few days), MMP-9 damages the blood-brain barrier, causing leakage, leukocyte infiltration, cerebral edema, and even bleeding [44]. MMP-9 levels are closely associated with the degree of neurological deficit and infarct volume in patients with acute cerebral infarction [45]. Elevated MMP-9 levels can be used as predictor of poor prognosis and stroke-related death [46]. A previous study showed that Kudiezi injection alleviated the dysfunction of the blood-brain barrier in rats with cerebral ischemia through the inhibition of the degradation of a number of proteins [18]; unfortunately, MMPs were not assessed. The present study showed that MMP-9 level started to decline from day 3 after admission in the KDZ group and that the MMP-9 levels were lower than in the control group.

IL-10 is an anti-inflammatory cytokine synthesized in the central nervous system. IL-10 reduces IL-1 and TNF-*α* production by inhibiting the expression and activation of cytokine receptors, and IL-10 has a protective effect in ischemic injuries [36]. In addition, low levels of peripheral serum IL-10 can increase the risk of stroke [35]. IL-10 levels in patients with acute cerebral infarction can increase at the initial stage of stroke, and IL-10 levels are positively correlated with disease severity, which is in line with the results of the present study. Indeed, IL-10 levels in patients with acute cerebral infarction at admission were higher than the normal reference values. After 3 days of Kudiezi injection, IL-10 levels increased significantly, but there was no difference with the control group. This is supported by a previous study in rats [19].

The present study is not without limitations. Only levels of various serum inflammatory factors were measured, and other pathological processes involved in ischemic damage (e.g., oxidative stress) were not assessed. A previous study in rats showed that Kudiezi injection could decrease oxidative stress after stroke, as well as secondary myocardial damage in rats [25]. This aspect could be worth exploring in humans. In addition, the mid- and long-term levels of the serum inflammatory markers after Kudiezi injection treatment are unknown. The small sample size may also affect the statistical results. Finally, the NIHSS could be not sensitive enough to detect differences in neurological function between the two groups. Additional studies are needed to overcome these limitations.

## 5. Conclusion

Taken together, those results suggested that Kudiezi injection could lead to early reduction of IL-6, IL-18, MMP-9, NSE, and S100B levels and increase of IL-10 levels in patients with acute ischemic stroke. Future studies will be performed in larger samples of patients and from different regions of China in order to improve generalizability. Additional biomarkers will also be assayed in order to uncover the exact mechanisms of Kudiezi injection in patients with stroke. Moreover, further analysis about the correlations between inflammatory biomarkers and NIHSS scores will be conducted. Longer-term follow-up will also be performed to examine the effects of Kudiezi injection on stroke recurrence and mortality.

## Figures and Tables

**Table 1:** Baseline characteristic of the patients.

	Controls	KDZ	*P*
*n*	28	28	
Age, mean (years)	62.0 ± 12.1	62.2 ± 16.0	0.963
Gender (F : M)	11 : 17	10 : 18	1.000
Risk factors, *n* (%)			
Hypertension	15 (53.6)	17 (60.7)	0.787
Diabetes	9 (32.1)	9 (32.1)	1.000
Hypercholesterolemia	9 (32.1)	7 (25.0)	0.554
Dyslipidemia	9 (32.1)	7 (25.0)	0.768
Stroke history	6 (21.4)	4 (14.3)	0.729
Smoking	27 (96.4)	28 (100.0)	0.313
Complications, *n* (%)			
Chronic kidney disease	1 (3.6)	2 (7.1)	1.000
Chronic lung disease	2 (7.1)	1 (3.6)	1.000
Coronary heart disease	5 (17.9)	3 (10.7)	0.705
Atrial fibrillation	1 (3.6)	1 (3.6)	1.000
Valvular heart disease/peripheral arterial disease	0/0	0/0	—
Stroke subtypes, *n* (%)			
Large-artery atherosclerosis	3 (10.7)	1 (3.6)	
Small blood vessel occlusion	12 (42.9)	14 (50.0)	
Other or unknown causes	13 (46.4)	13 (46.4)	
